# Development and validation of a prediction model for postoperative cerebral edema progression in emergency traumatic brain injury patients

**DOI:** 10.1097/MD.0000000000048382

**Published:** 2026-05-15

**Authors:** Fengchao Zhang, Jianfang Zheng, Yan Liu, Huifang Lin

**Affiliations:** a Emergency Department, Xingtai People’s Hospital, Xingtai City, Hebei Province, China.

**Keywords:** logistic regression, model validation, postoperative cerebral edema, prediction model, risk factors, traumatic brain injury

## Abstract

To develop and internally validate a clinical prediction model for estimating the risk of postoperative cerebral edema progression in emergency traumatic brain injury (TBI) patients. This study was a single-center, retrospective, observational cohort study. A total of 201 TBI patients who were admitted to our hospital’s emergency department and underwent craniotomy between January 2023 and December 2024 were consecutively enrolled. Based on imaging and clinical criteria within 72 hours postoperatively, patients were categorized into a cerebral edema progression group (n = 63) and a non-progression group (n = 138). Using the time-split method, 70% of the patients (n = 141) were assigned to the model development cohort, and 30% (n = 60) to the internal validation cohort. Demographic, clinical, imaging, and laboratory indicators were collected via systematic review of electronic medical records. In the development cohort, univariate logistic regression was first performed to screen potential predictors (*P* < .1), followed by multivariate logistic regression to identify independent predictors and build the prediction model. Model performance was assessed by the area under the receiver operating characteristic curve (AUC) for discrimination, the Hosmer–Lemeshow test and calibration plot for calibration, and decision curve analysis for clinical utility. Finally, subgroup analysis was conducted to verify the model’s stability. Multivariate analysis ultimately identified 7 independent predictors of postoperative cerebral edema progression: increased age (adjusted odds ratio [aOR] = 1.02/yr), lower admission Glasgow Coma Scale score (aOR = 0.86/point), larger volume of cerebral contusion/hematoma (aOR = 1.06/10 mL), greater midline shift (aOR = 1.12/mm), higher maximum intracranial pressure within 24 hours postoperatively (aOR = 1.11/mm Hg), lower serum sodium level within 24 hours postoperatively (aOR = 0.91/mmol/L), and elevated C-reactive protein level (aOR = 1.03/10 mg/L). The prediction model constructed based on these factors demonstrated excellent discrimination in the development cohort (AUC = 0.901, 95% CI: 0.849–0.954) and maintained good predictive performance in the internal validation cohort (AUC = 0.875, 95% CI: 0.782–0.948). This study successfully developed and internally validated a prediction model incorporating clinical, imaging, and laboratory indicators. This model can accurately and stably predict the risk of postoperative cerebral edema progression in emergency TBI patients, facilitating the early identification of high-risk patients and guiding individualized intervention.

## 1. Introduction

Traumatic brain injury (TBI) is one of the leading global causes of death and disability, imposing a substantial burden on society.^[1,2]^ For patients with severe TBI admitted to the emergency department, emergency craniotomy (such as decompressive craniectomy or intracranial hematoma evacuation) is a critical life-saving measure to relieve cerebral herniation and reduce intracranial pressure.^[3,4]^ However, the surgery itself, as a secondary insult, often induces or exacerbates perioperative cerebral edema, leading to the progression of postoperative cerebral edema. This is a significant complication contributing to clinical deterioration and poor outcomes in these patients.^[5,6]^

Progressive postoperative cerebral edema can cause a recurrent increase in intracranial pressure, potentially triggering secondary cerebral herniation, severely impairing neurological recovery, and increasing mortality.^[7]^ Although clinicians can monitor patients closely through serial head computed tomography [CT] scans and intracranial pressure monitoring, the progression of cerebral edema is often only detected when obvious clinical symptoms or radiological signs appear, by which time the optimal “therapeutic window” for intervention might have been missed.^[8]^ Therefore, the early postoperative identification of high-risk patients, even before clear clinical or radiological signs of edema progression emerge, is crucial for enabling early warning and initiating targeted intensive therapies (such as optimized sedation, precise hyperosmolar therapy, or prophylactic hypothermia).^[9]^

Current research has explored various risk factors associated with post-traumatic cerebral edema, including advanced age, low admission Glasgow Coma Scale (GCS) score, large initial hematoma volume, coagulopathy, and systemic inflammatory response.^[10,11]^ However, these factors are often assessed in isolation or a fragmented manner, lacking an integrated predictive tool that combines patient demographics, injury severity, imaging characteristics, and early pathophysiological responses (such as inflammation and metabolic disturbances). A rigorously validated prediction model capable of quantifying individual risk would significantly aid clinical decision-making and optimize resource allocation.

Based on this rationale, this study aims to systematically develop and internally validate a clinical prediction model for estimating the risk of postoperative cerebral edema progression in emergency TBI patients, through a single-center, retrospective, observational cohort study. We anticipate that this model will provide clinicians with an objective decision-support tool usable early in the postoperative period, thereby informing the individualized management of high-risk patients.

## 2. Materials and methods

### 2.1. Study design

This study was approved by the Ethics Committee of Xingtai People’s Hospital. This study was a single-center, retrospective, observational cohort study. The research plan involved the consecutive enrollment of TBI patients who were admitted to our hospital’s emergency department and underwent craniotomy between January 2023 and December 2024. The objective was to develop and internally validate a clinical model for predicting the risk of postoperative cerebral edema progression based on this cohort. Ultimately, a total of 201 consecutive cases meeting the predefined inclusion and exclusion criteria were included to ensure sufficient statistical power for model development and validation.

### 2.2. Inclusion and exclusion criteria

#### 2.2.1. Inclusion criteria

Age ≥18 years; clinical and radiological confirmation of TBI; underwent emergency craniotomy within 24 hours of admission; and underwent a baseline head CT scan within 6 hours postoperatively and at least one follow-up CT scan between 24 and 72 hours postoperatively.

#### 2.2.2. Exclusion criteria

History of significant intracranial pathology (e.g., brain tumor, large-area cerebral infarction, hydrocephalus, etc); combined with severe life-threatening trauma to other systems (e.g., thoracic, abdominal); missing clinical or radiological data leading to unreliable outcome assessment; and death or automatic discharge from the hospital within 72 hours postoperatively.

### 2.3. Grouping

To establish and internally validate the prediction model, patients were divided using a time-split method based on chronological order: ~70% of the patients (n = 141, including 45 in the progression group and 96 in the non-progression group) enrolled during the earlier period were assigned to the model development cohort for variable screening and model construction; subsequently, approximately 30% of the patients (n = 60, including 18 in the progression group and 42 in the non-progression group) enrolled during the later period were assigned to the model validation cohort for internal validation of model performance, including assessments of discrimination and calibration.

Based on follow-up imaging results and clinical records within 72 hours postoperatively, all patients were further classified into a cerebral edema progression group and a non-progression group. The criteria for determining cerebral edema progression were defined as any of the following changes on the CT scan performed between 24 and 72 hours postoperatively compared to the postoperative baseline CT (performed within ≤6 hours postoperatively):

① Midline shift increased by ≥2 mm from baseline;
② Sulcal or ventricular effacement worsened from baseline (semi-quantitative score increased by ≥1 grade);
③ The volume of the low-density edema area increased by ≥25% from baseline;

Or the requirement for intensified therapy (such as sustained hyperosmolar therapy, external ventricular drainage, or secondary decompressive surgery) due to elevated intracranial pressure or radiological deterioration within 72 hours postoperatively.

Patients who did not exhibit any of the above changes were classified into the non-progression group.

Imaging assessments were independently performed by 2 physicians experienced in neuroimaging under blinded conditions. In case of disagreement, a third researcher would review and adjudicate the findings to ensure objectivity and consistency in outcome determination.

### 2.4. Data collection

Through systematic review of the hospital’s electronic medical record system, we comprehensively collected clinical data relevant to the study. The collected content encompassed demographic characteristics, baseline clinical information, imaging data, laboratory test indicators, as well as treatment processes and outcome variables. All data collection was performed using standardized forms and completed independently by 2 researchers who conducted cross-checking to ensure accuracy and completeness of the information.

Regarding demographic and baseline clinical data, we focused on recording the patient’s sex, age, injury mechanism, and GCS score at admission. Surgical-related indicators were also collected in detail, including the specific surgical approach, duration of surgery, and intraoperative blood loss, which reflect the extent and complexity of surgical trauma.

For imaging evaluation, assessments were based on the baseline head CT scans completed within 6 hours postoperatively. These were independently evaluated by 2 neuroradiologists blinded to the group allocation. Evaluation content included the volume of cerebral contusion/hematoma, status of the basal cisterns, presence or absence of traumatic subarachnoid hemorrhage or intraventricular hemorrhage, and the distance of midline shift. In cases of disagreement between the 2 evaluators, a third senior researcher arbitrated to ensure objectivity and accuracy of the imaging assessments.

The collection of laboratory and physiological monitoring indicators covered multiple dimensions of pathophysiological changes. We collected indicators reflecting coagulation function, including prothrombin time, activated partial thromboplastin time, international normalized ratio, fibrinogen, and d-dimer. Indicators related to inflammatory response were also recorded, such as white blood cell (WBC) count, neutrophil percentage, C-reactive protein, and procalcitonin. For metabolic indicators, we collected blood glucose levels at admission and 24 hours postoperatively, as well as blood lactate levels. Additionally, the maximum intracranial pressure and serum sodium levels within the first 24 hours postoperatively were recorded, reflecting the patient’s intracranial status and internal environment.

Intracranial pressure (ICP) was continuously monitored postoperatively in patients with invasive monitoring. The maximum (peak) ICP value recorded within the first 24 hours after surgery was used for analysis. Serum sodium and blood glucose levels were obtained from routine postoperative laboratory tests, and the lowest serum sodium value and highest blood glucose value within 24 hours postoperatively were recorded.

C-reactive protein (CRP) levels were measured using standardized laboratory assays, with samples collected during routine postoperative blood testing within 24 hours after surgery. When multiple measurements were available within this period, the highest CRP value was used for analysis. All laboratory data were extracted following uniform clinical protocols to minimize measurement variability and improve model stability.

Finally, regarding treatment and outcomes, we detailed whether patients received hyperosmolar therapy postoperatively, the length of stay in the intensive care unit, and strictly determined whether postoperative cerebral edema progression occurred according to the predefined imaging and clinical criteria. This systematic collection of data provided a comprehensive information base for the subsequent development and validation of the model.

### 2.5. Statistical analysis

Statistical analysis was performed using SPSS 26.0 and R 4.2.1 software. Continuous variables conforming to a normal distribution are expressed as mean ± standard deviation and were compared between groups using the *t*-test; non-normally distributed variables are expressed as median (interquartile range) and were compared using the Mann–Whitney *U* test. Categorical variables are expressed as number (%) and were compared using the χ^2^ test or Fisher’s exact test. The missing data rate for each variable in this study was below 5%. Random missing data in continuous variables were handled using multiple imputation, while missing data in categorical variables led to the exclusion of the respective observations.

In the model development cohort (n = 141), univariate logistic regression analysis was first performed (with *P* < .1 as the screening criterion). Subsequently, the screened variables were incorporated into a multivariate logistic regression model, and independent predictors were identified using the forward likelihood ratio method to establish the prediction model. Model performance was evaluated by the area under the receiver operating characteristic curve (AUC) for discrimination, the Hosmer–Lemeshow test for calibration, and decision curve analysis for clinical utility. Finally, subgroup analysis was conducted to validate the stability of the model across different patient populations. All tests were 2-sided, and a *P*-value < .05 was considered statistically significant.

## 3. Results

### 3.1. Patient baseline characteristics

A total of 201 patients were included in the analysis, of whom 63 (31.3%) developed postoperative cerebral edema progression. A comparison of the baseline characteristics between the 2 groups is presented in Table 1. Compared to the non-progression group, patients in the progression group were older, had a lower admission GCS score, and had a longer operative time (all *P* < .05). Regarding preoperative imaging, the progression group had a larger volume of cerebral contusion/hematoma, a higher incidence of compressed/obliterated basal cisterns and traumatic subarachnoid hemorrhage/intraventricular hemorrhage, and a more significant midline shift (all *P* < .01). In terms of postoperative management, the progression group had a higher maximum 24-hour ICP, lower 24-hour serum sodium levels, and received hyperosmolar therapy more frequently (all *P* < .01).

**Table 1:** Baseline characteristics of patients with acute traumatic brain injury after emergency craniotomy.

Variable	Total (n = 201)	Edema progression (n = 63)	Non-progression (n = 138)	Statistic	*P*-value
Sex (n [%])					
Male	156 (77.6)	51 (81.0)	105 (76.1)	χ^2^ = 0.62	.432
Female	45 (22.4)	12 (19.0)	33 (23.9)		
Age (yr, median [IQR])	48 (35–62)	56 (41–67)	44 (32–58)	*Z* = 2.89	.004
Admission GCS (median [IQR])	7 (5–10)	6 (4–8)	8 (6–11)	*Z* = −3.16	.002
Mechanism of injury (n [%])					
Traffic accident	112 (55.7)	39 (61.9)	73 (52.9)	χ^2^ = 1.42	.233
Fall	54 (26.9)	14 (22.2)	40 (29.0)		
Violence/others	35 (17.4)	10 (15.9)	25 (18.1)		
Surgical type [n (%)]					
Decompressive craniectomy	142 (70.6)	50 (79.4)	92 (66.7)	χ^2^ = 3.12	.077
Hematoma evacuation only	59 (29.4)	13 (20.6)	46 (33.3)		
Operation time (min, median [IQR])	185 (145–240)	205 (160–260)	175 (140–220)	*Z* = 2.56	.01
Preoperative imaging features					
Contusion/hematoma volume (mL, median [IQR])	35 (22–55)	48 (30–68)	30 (20–45)	*Z* = 4.01	<.001
Basal cistern compression/absence [n (%)]	85 (42.3)	38 (60.3)	47 (34.1)	χ^2^ = 12.34	<.001
Traumatic SAH/IVH [n (%)]	121 (60.2)	47 (74.6)	74 (53.6)	χ^2^ = 8.12	.004
Midline shift (mm, mean ± SD)	6.8 ± 3.7	8.2 ± 3.9	6.1 ± 3.4	*t* = 3.67	<.001
Intraoperative blood loss (mL, median [IQR])	350 (200–600)	380 (220–620)	340 (190–590)	*Z* = 1.02	.307
Post-operative 24 h peak ICP (mm Hg, mean ± SD)	23.6 ± 7.4	28.2 ± 8.1	21.4 ± 6.2	*t* = 6.09	<.001
Post-operative 24 h serum sodium (mmol/L, mean ± SD)	141.6 ± 5.3	139.8 ± 4.9	142.4 ± 5.3	*T* = −3.12	.002
Hyperosmolar therapy (n [%])	98 (48.8)	42 (66.7)	56 (40.6)	χ^2^ = 11.05	.001
ICU stay (d, median [IQR])	8 (5–13)	11 (7–16)	7 (4–11)	*Z* = 3.76	<.001

GCS = Glasgow Coma Scale, ICP = intracranial pressure, IVH = intraventricular hemorrhage, SAH = subarachnoid hemorrhage, SD = standard deviation.

### 3.2. Comparison of coagulation, inflammatory, and metabolic indicators

Comparison of laboratory indicators (Table 2) revealed more severe systemic pathophysiological alterations in the cerebral edema progression group. This group exhibited more significant coagulation dysfunction, manifested as higher prothrombin time, activated partial thromboplastin time, international normalized ratio, and d-dimer levels (all *P* < .05). Concurrently, the systemic inflammatory response was stronger, with significant elevations in WBC, neutrophil percentage, CRP, and procalcitonin (all *P* < .01). Metabolically, the progression group also had persistently higher blood glucose levels from admission to 24 hours postoperatively, as well as higher postoperative blood lactate levels (all *P* < .01).

**Table 2:** Comparison of coagulation function, inflammatory markers, and glucose metabolism between groups.

Variable	Total (n = 201)	Edema progression (n = 63)	Non-progression (n = 138)	Statistic	*P* value
Coagulation parameters					
PT (s, mean ± SD)	13.7 ± 1.9	14.2 ± 2.1	13.5 ± 1.8	*t* = 2.34	.02
APTT (s, mean ± SD)	36.8 ± 5.9	38.4 ± 6.1	36.1 ± 5.7	*t* = 2.52	.013
INR (mean ± SD)	1.13 ± 0.14	1.18 ± 0.16	1.11 ± 0.13	*t* = 2.88	.004
Fibrinogen (g/L, mean ± SD)	3.62 ± 0.96	3.48 ± 0.92	3.69 ± 0.97	*t* = −1.45	.149
d-dimer (mg/L, median [IQR])	2.4 (1.1–4.8)	3.3 (1.6–6.0)	2.0 (1.0–3.9)	*Z* = 3.16	.002
Inflammatory markers					
WBC (×10^9^/L, mean ± SD)	13.2 ± 4.5	14.8 ± 4.9	12.5 ± 4.1	*t* = 3.25	.001
Neutrophil ratio (%, mean ± SD)	81.5 ± 8.9	84.2 ± 8.1	80.3 ± 9.1	*t* = 2.84	.005
CRP (mg/L, median [IQR])	48.6 (22.5–96.8)	72.3 (35.4–122.7)	39.8 (19.1–78.5)	*Z* = 3.67	<.001
PCT (ng/mL, median [IQR])	0.24 (0.10–0.58)	0.35 (0.15–0.81)	0.20 (0.09–0.44)	*Z* = 2.98	.003
Glucose metabolism					
Admission glucose (mmol/L, mean ± SD)	8.4 ± 2.3	9.3 ± 2.6	8.0 ± 2.1	*t* = 3.33	.001
Post-operative 24 h glucose (mmol/L, mean ± SD)	8.9 ± 2.5	9.8 ± 2.8	8.5 ± 2.3	*t* = 3.04	.003
Post-operative 24 h lactate (mmol/L, median [IQR])	2.3 (1.6–3.5)	2.8 (2.0–3.9)	2.1 (1.4–3.2)	*Z* = 2.85	.004

APTT = activated partial thromboplastin time, CRP = C-reactive protein, INR = international normalized ratio, PCT = procalcitonin, PT = prothrombin time, SD = standard deviation, WBC = white blood cell.

### 3.3. Screening of predictors for postoperative cerebral edema progression by univariate logistic regression analysis

In the model development cohort (n = 141), univariate logistic regression analysis was performed on all potential predictor variables to preliminarily identify factors associated with postoperative cerebral edema progression. The detailed results of the analysis are presented in Table 3.

**Table 3:** Univariate logistic regression for predictors of postoperative cerebral edema progression in the development cohort (n = 141; events = 45).

Variable	β	OR	95% CI	*P*-value
Male sex (vs female)	0.22	1.25	0.63–2.46	.52
Age (per 1 year increase)	0.028	1.03	1.01–1.06	.018
Admission GCS (per 1-point increase)	−0.165	0.85	0.74–0.97	.017
Traffic accident (vs violence/others)	0.32	1.38	0.73–2.62	.32
Fall (vs violence/others)	0.06	1.06	0.51–2.20	.88
Decompressive craniectomy (vs hematoma evacuation)	0.46	1.58	0.84–3.00	.16
Operation time (per 10 min increase)	0.036	1.04	1.01–1.08	.024
Intraoperative blood loss (per 100 mL increase)	0.041	1.04	0.95–1.15	.4
Contusion/hematoma volume (per 10 mL increase)	0.078	1.08	1.03–1.14	.002
Basal cistern compression/absence (yes vs no)	0.89	2.43	1.25–4.73	.009
Traumatic SAH/IVH (yes vs no)	0.69	2	1.10–3.64	.022
Midline shift (per 1 mm increase)	0.16	1.17	1.06–1.30	.002
Peak ICP (per 1 mm Hg increase)	0.138	1.15	1.07–1.24	<.001
Serum sodium (per 1 mmol/L increase)	−0.105	0.9	0.82–0.99	.036
Hyperosmolar therapy (yes vs no)	0.81	2.25	1.16–4.36	.016
ICU stay (per 1 day increase)	0.071	1.07	1.02–1.13	.008
PT (per 1 s increase)	0.185	1.2	0.99–1.46	.062
APTT (per 1 s increase)	0.058	1.06	1.00–1.12	.049
INR (per 0.1 increase)	1.49	2.23	1.01–4.95	.047
Fibrinogen (per 1 g/L increase)	−0.120	0.89	0.72–1.10	.28
d -dimer (per 1 mg/L increase)	0.161	1.17	1.02–1.35	.027
WBC (per 1 × 10^9^/L increase)	0.081	1.08	1.01–1.16	.03
Neutrophil ratio (per 1% increase)	0.044	1.05	1.01–1.09	.017
CRP (per 10 mg/L increase)	0.033	1.03	1.01–1.06	.006
PCT (per 0.1 ng/mL increase)	0.238	1.27	1.05–1.54	.014
Admission glucose (per 1 mmol/L increase)	0.198	1.22	1.05–1.42	.01
Post-operative 24 h glucose (per 1 mmol/L increase)	0.166	1.18	1.03–1.36	.02
Post-operative 24 h lactate (per 1 mmol/L increase)	0.245	1.28	1.06–1.55	.011

APTT = activated partial thromboplastin time, CI = confidence interval, CRP = C-reactive protein, GCS = Glasgow Coma Scale, ICP = intracranial pressure, INR = international normalized ratio, IVH = intraventricular hemorrhage, OR = odds ratio, PCT = procalcitonin, PT = prothrombin time, SAH = subarachnoid hemorrhage, SD = standard deviation, WBC = white blood cell.

### 3.4. Identification of independent predictors for postoperative cerebral edema progression by multivariate logistic regression and development of the prediction model

To identify independent predictors of postoperative cerebral edema progression and develop a robust prediction model, variables with a *P*-value < .1 from the univariate analysis were included in a multivariate logistic regression analysis. Given the limited number of events (n = 45) in the development cohort, and to adhere to the statistical principle of at least 10 events per variable to avoid model overfitting, variables were selected based on statistical significance and clinical relevance: variables with high collinearity (e.g., choosing representative indicators between WBC and CRP, and between admission blood glucose and 24-hour postoperative blood glucose) were excluded. After stepwise selection, 7 key variables were ultimately included and confirmed as independent predictors, and the prediction model was constructed based on these. The results of the multivariate analysis are detailed in Table 4. Based on these factors, the resulting prediction model formula is:

**Table 4:** Multivariate logistic regression analysis for independent predictors of postoperative cerebral edema progression in the development cohort (n = 141).

Variable	β	Adjusted OR	95% CI	*P*-value
Age (per 1 year increase)	0.024	1.02	1.00–1.05	.041
Admission GCS (per 1-point increase)	−0.152	0.86	0.75–0.98	.025
Contusion/hematoma volume (per 10 mL increase)	0.061	1.06	1.01–1.12	.017
Midline shift (per 1 mm increase)	0.114	1.12	1.02–1.25	.021
Post-operative 24 h peak ICP (per 1 mm Hg increase)	0.102	1.11	1.04–1.19	.002
Serum sodium (per 1 mmol/L increase)	−0.097	0.91	0.83–0.99	.032
CRP (per 10 mg/L increase)	0.027	1.03	1.00–1.05	.045

CI = confidence interval, CRP = C-reactive protein, GCS = Glasgow Coma Scale, ICP = intracranial pressure, OR = odds ratio.

**Figure 1.**
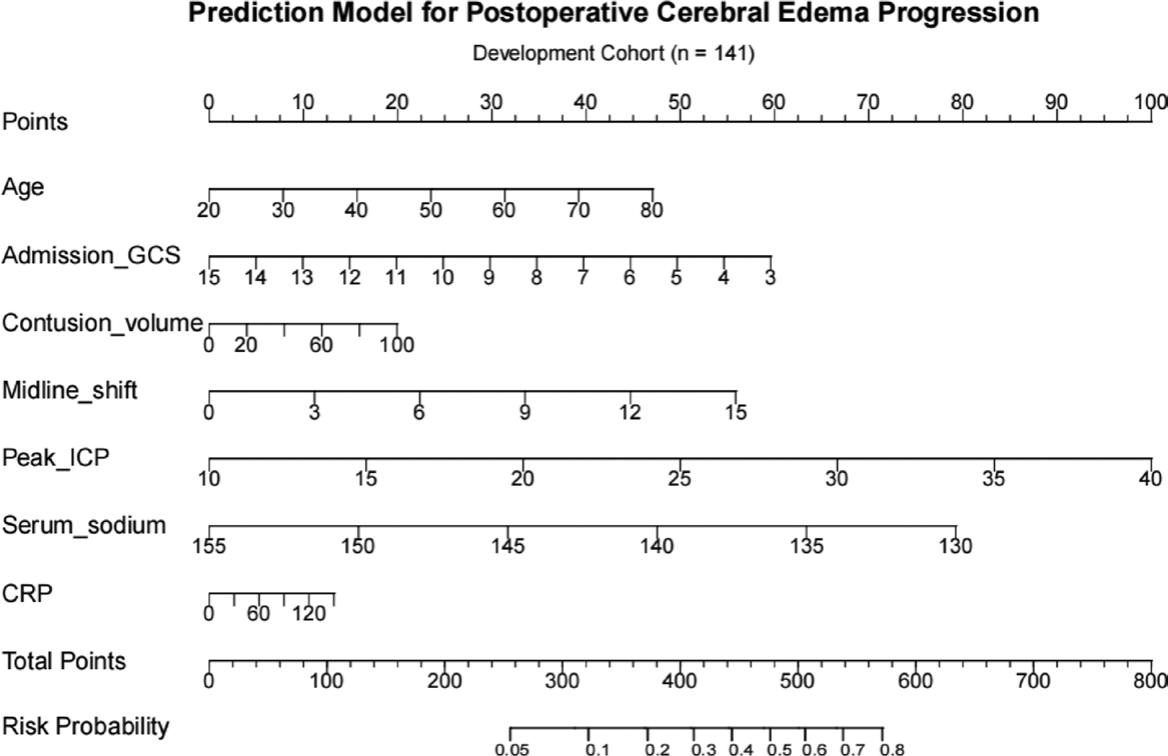
Risk prediction model lollipop chart.

Logit(*P*) = −7.214 + 0.024 × age − 0.152 × GCS + 0.061 × Hematoma volume (per 10 mL) + 0.114 × midline shift (mm) + 0.102 × peak ICP (mm Hg) − 0.097 × serum sodium (mmol/L) + 0.027 × CRP (per 10 mg/L). Based on this, nomogram 1 was constructed. Each of these variables was assigned an individual score, ranging from 0 to 100. Furthermore, the scores of all variables were summed to generate a total score, which corresponds to the probability of progressive cerebral edema (Fig. 1).

### 3.5. Performance validation of the prediction model

**Figure 2.**
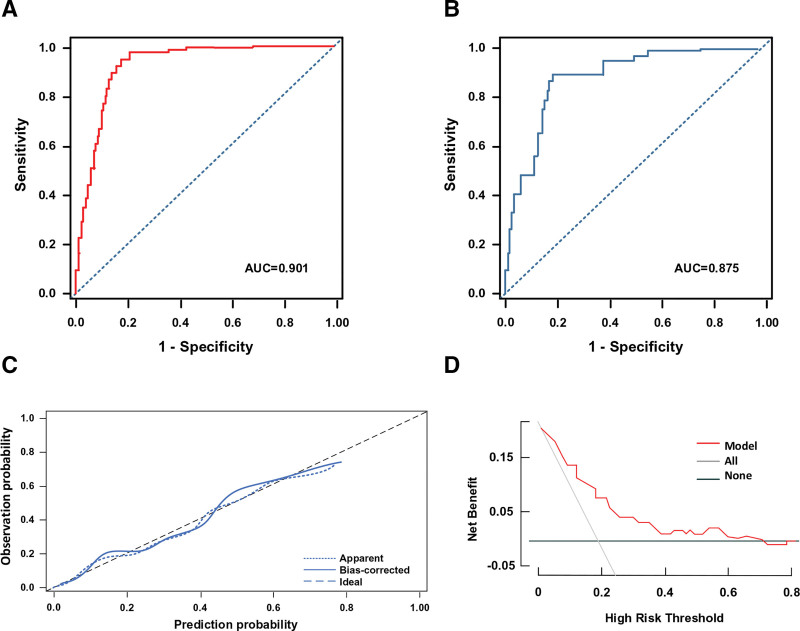
Evaluation of the predictive model’s capabilities. (A) ROC curve of the development cohort; (B) ROC curve of the validation cohort; (C) calibration curve; and (D) DCA curve. ROC = area under the curve.

The model demonstrated excellent performance in both the development and validation cohorts (Table 5). The AUC was 0.901 in the development cohort (Fig. 2A) and 0.875 in the validation cohort (Fig. 2B), indicating outstanding discriminative ability and good generalizability of the model. At the optimal cutoff value, the model’s sensitivity and specificity both exceeded 80% in both cohorts. The Hosmer–Lemeshow test yielded *P*-values >.05, suggesting good calibration between the model’s predicted probabilities and the actual observed incidence. The calibration plot (Fig. 2C) showed that the predictions from the nomogram model were in good agreement with the actual observations. According to the decision curve analysis (Fig. 2D), the net benefit of the prediction model for the internal validation set was significantly higher than the 2 extreme scenarios, indicating that the nomogram model possesses superior net benefit and predictive accuracy.

**Table 5:** ROC analysis of the predictive model for postoperative cerebral edema progression.

Cohort	AUC (95% CI)	Sensitivity (%)	Specificity (%)	Youden index	Optimal cutoff (*P* cutoff)	Hosmer–Lemeshow χ^2^ (*P*-value)
Development cohort (n = 141)	0.901 (0.849–0.954)	82.2	84.6	0.667	.42	6.72 (.57)
Validation cohort (n = 60)	0.875 (0.782–0.948)	80	81	0.61	.45	5.98 (.64)

AUC = area under the curve, CI = confidence interval.

### 3.6. Subgroup analysis of the prediction model

Subgroup analysis demonstrated that the prediction model performed consistently and excellently across all key clinical subgroups (Table 6). Specifically, the model maintained high predictive efficacy in patients with different surgical approaches (decompressive craniectomy AUC = 0.903, hematoma evacuation AUC = 0.887), different injury mechanisms (traffic accident AUC = 0.898, fall injury AUC = 0.883), different age stratifications (≥60 years AUC = 0.892, <60 years AUC = 0.905), and different disease severities (GCS ≤ 8 AUC = 0.908, GCS > 8 AUC = 0.879). The differences in AUC between the various subgroups were not statistically significant (all *P* > .05; Fig. 2).

**Table 6:** Subgroup analysis of model performance for postoperative cerebral edema progression.

Subgroup	n	Edema progression, n (%)	AUC (95% CI)	*P* AUC_diff
Surgical type				
Decompressive craniectomy	142	50 (35.2)	0.903 (0.848–0.957)	.47
Hematoma evacuation only	59	13 (22.0)	0.887 (0.802–0.946)	
Mechanism of injury				
Traffic accident	112	39 (34.8)	0.898 (0.835–0.952)	.58
Fall	54	14 (25.9)	0.883 (0.796–0.943)	
Age group (yr)				
≥60	64	26 (40.6)	0.892 (0.811–0.951)	.64
<60	137	37 (27.0)	0.905 (0.849–0.956)	
Admission GCS				
≤8	122	45 (36.9)	0.908 (0.854–0.959)	.42
>8	79	18 (22.8)	0.879 (0.796–0.940)	

AUC = area under the curve, CI = confidence interval, GCS = Glasgow Coma Scale.

## 4. Discussion

This study successfully developed and internally validated a clinical prediction model for assessing the risk of postoperative cerebral edema progression, based on a cohort of 201 acute TBI patients undergoing emergency craniotomy. The model integrates 7 readily obtainable clinical, imaging, and laboratory indicators – age, admission GCS score, cerebral contusion/hematoma volume, midline shift, maximum intracranial pressure (ICP) within 24 hours postoperatively, serum sodium level within 24 hours postoperatively, and C-reactive protein (CRP) – and presents them intuitively via a nomogram. Validation results demonstrated that the model possesses excellent discriminatory power (AUC = 0.901 in the development cohort; AUC = 0.875 in the validation cohort), satisfactory calibration, and confirmed clinical net benefit, while maintaining stable performance across key subgroups. This provides clinicians with a robust quantitative tool for the early postoperative identification of high-risk patients and the implementation of proactive interventions.

The independent predictors selected by our model have a clear pathophysiological basis. Advanced age is typically associated with reduced brain tissue compliance, impaired cerebrovascular autoregulation, and diminished tolerance to secondary injury, establishing it as a recognized risk factor for various adverse neurological outcomes.^[12]^ A low admission GCS score directly reflects the severity of the primary brain injury and serves as a cornerstone for the progression of subsequent secondary insults.^[13]^ Larger cerebral contusion/hematoma volumes and significant midline shift are objective imaging markers of intracranial mass effect and mechanical injury severity. They not only cause direct parenchymal damage but also initiate a cascade of complex pathophysiological responses – including local cerebral perfusion disturbances, blood–brain barrier disruption, and inflammatory activation – that drive a vicious cycle of cerebral edema.^[5,14]^ Early postoperative elevation of ICP is both a direct consequence and a core clinical manifestation of deteriorating intracranial dynamics due to progressive edema; its inclusion in the model substantially enhances predictive timeliness and accuracy.^[15]^

Of particular note, this study underscores the critical value of systemic pathophysiological alterations in prediction. Hyponatremia, a common electrolyte disturbance following TBI, can exacerbate vasogenic cerebral edema through osmotic mechanisms. Its presence often indicates hypothalamic-pituitary axis involvement or the occurrence of syndrome of inappropriate antidiuretic hormone secretion.^[16]^ C-reactive protein (CRP), as a classic systemic inflammatory marker, demonstrated a positive correlation between its elevated levels and model risk, strongly supporting the theory that “systemic inflammatory response participates in and exacerbates local cerebral edema after traumatic brain injury.”^[17,18]^ While previous studies have predominantly focused on local injury or intracranial parameters, our model emphasizes the importance of monitoring patients’ systemic internal environment and immune status in predicting postoperative cerebral edema by integrating serum sodium and CRP, thereby providing a new “local-systemic” multidimensional perspective for understanding this complication. Although the predictors included in the model are biologically plausible and supported by previous studies, it should be noted that this prediction model is not intended to establish causal relationships. Variables such as intracranial pressure, serum sodium, and C-reactive protein likely reflect the overall severity of secondary injury and systemic stress responses rather than direct mechanistic pathways of cerebral edema progression. Therefore, the clinical interpretation of individual predictors should be made with caution, and the model should be used primarily as a risk stratification tool rather than a mechanistic explanation framework.

In terms of model construction and methodology, this study maintained rigorous standards. We employed a time-split method for internal validation, which more effectively simulates the model’s generalizability in real-world clinical temporal flow compared to simple random splitting.^[19]^ We strictly adhered to statistical guidelines for prediction model research, considering the events-per-variable principle during variable selection and utilizing stepwise regression to avoid overfitting.^[20]^ The final model not only demonstrated high discriminative ability but also passed the Hosmer–Lemeshow test and calibration curve evaluation, confirming strong agreement between predicted probabilities and actual observed risks. Decision curve analysis further demonstrated that across a wide range of threshold probabilities, the net benefit of using this model to guide clinical decisions substantially exceeded both the “treat-all” and “treat-none” strategies, highlighting its clinical utility.^[21]^ Comprehensive subgroup analysis results reinforced the model’s robustness, indicating that its predictive performance remains consistent regardless of surgical approach, injury mechanism, age, or baseline disease severity, demonstrating its potential for application in heterogeneous patient populations.

Certainly, this study has several limitations. First, this was a single-center, retrospective study, and although internal validation demonstrated good discrimination and calibration, external validation using independent, multi-center cohorts was not performed. Differences in patient management strategies, surgical indications, perioperative monitoring protocols, and resource availability across institutions may influence model performance. Therefore, the generalizability of this prediction model to other centers and healthcare systems remains uncertain. Future prospective, multi-center studies are warranted to externally validate and, if necessary, recalibrate the model before its widespread clinical application. Second, although we included various indicators such as coagulation and inflammatory markers, some potential predictors – such as more specific neurobiomarkers (e.g., glial fibrillary acidic protein, ubiquitin C-terminal hydrolase-L1) or advanced imaging parameters (e.g., diffusion tensor imaging) – were not included in the analysis due to the lack of routine testing in this retrospective cohort. These could represent directions for future model refinement.^[22]^ Finally, the number of cerebral edema progression events in the model development cohort was 45. Although we controlled model complexity through strict variable selection strategies, a larger sample size would allow for the exploration of more potential predictor variables and the construction of a more robust model.

In summary, this study developed and validated a comprehensive prediction model capable of accurately and individually estimating the risk of postoperative cerebral edema progression in emergency TBI patients. The model is based on routinely available clinical parameters, demonstrates strong operational feasibility, and exhibits good discrimination, calibration, and clinical utility. Once validated in broader populations, this model holds promise as an effective tool to assist clinicians in early risk stratification, optimization of postoperative management strategies, and ultimately, improvement of patient outcomes.

## Author contributions

**Conceptualization:** Fengchao Zhang, Jianfang Zheng, Yan Liu, Huifang Lin.

**Data curation:** Fengchao Zhang, Jianfang Zheng, Yan Liu, Huifang Lin.

**Formal analysis:** Fengchao Zhang, Jianfang Zheng, Yan Liu, Huifang Lin.

**Funding acquisition:** Fengchao Zhang, Jianfang Zheng, Yan Liu.

**Investigation:** Fengchao Zhang, Jianfang Zheng, Yan Liu.

**Writing – original draft:** Fengchao Zhang, Jianfang Zheng, Huifang Lin.

**Writing – review & editing:** Fengchao Zhang, Jianfang Zheng, Huifang Lin.
